# Lasting EEG/MEG Aftereffects of Rhythmic Transcranial Brain Stimulation: Level of Control Over Oscillatory Network Activity

**DOI:** 10.3389/fncel.2015.00477

**Published:** 2015-12-15

**Authors:** Domenica Veniero, Alexandra Vossen, Joachim Gross, Gregor Thut

**Affiliations:** ^1^Centre for Cognitive Neuroimaging, Institute of Neuroscience and Psychology, University of GlasgowGlasgow, UK; ^2^School of Psychology, University of GlasgowGlasgow, UK

**Keywords:** human brain oscillations, offline aftereffects, transcranial alternating current stimulation, repetitive transcranial magnetic stimulation (rTMS), oscillatory transcranial direct current stimulation, entrainment, LTP/LTP-like plasticity

## Abstract

A number of rhythmic protocols have emerged for non-invasive brain stimulation (NIBS) in humans, including transcranial alternating current stimulation (tACS), oscillatory transcranial direct current stimulation (otDCS), and repetitive (also called rhythmic) transcranial magnetic stimulation (rTMS). With these techniques, it is possible to match the frequency of the externally applied electromagnetic fields to the intrinsic frequency of oscillatory neural population activity (“frequency-tuning”). Mounting evidence suggests that by this means tACS, otDCS, and rTMS can entrain brain oscillations and promote associated functions in a frequency-specific manner, in particular during (i.e., online to) stimulation. Here, we focus instead on the changes in oscillatory brain activity that persist after the end of stimulation. Understanding such aftereffects in healthy participants is an important step for developing these techniques into potentially useful clinical tools for the treatment of specific patient groups. Reviewing the electrophysiological evidence in healthy participants, we find aftereffects on brain oscillations to be a common outcome following tACS/otDCS and rTMS. However, we did not find a consistent, predictable pattern of aftereffects across studies, which is in contrast to the relative homogeneity of reported online effects. This indicates that aftereffects are partially dissociated from online, frequency-specific (entrainment) effects during tACS/otDCS and rTMS. We outline possible accounts and future directions for a better understanding of the link between online entrainment and offline aftereffects, which will be key for developing more targeted interventions into oscillatory brain activity.

## Introduction

Oscillatory brain activity is thought to reflect the assembly (i.e., synchronization) of neuronal elements into functional networks as an essential component of information processing (e.g., Buzsáki, 2006) and has been suggested to play a mechanistic role in normal function (e.g., Fries, 2005; Wang, 2010; Thut et al., 2012; Lopes da Silva, 2013) and dysfunction of the brain (e.g., Schnitzler and Gross, 2005; Uhlhaas and Singer, 2015). This has sparked an interest in specifically interacting with oscillatory activity by means of rhythmic non-invasive brain stimulation (NIBS). Protocols that are promising in this regard are transcranial alternating current stimulation (tACS), oscillatory transcranial direct current stimulation (otDCS), and repetitive/rhythmic transcranial magnetic stimulation (rTMS) (see Box 1). Despite the different mechanisms by which tACS/otDCS and rTMS exert their effect on the brain (weak electrical currents vs. stronger magnetic pulses: see Box 1), there is growing evidence that either technique can be used to impose a rhythmic structure on the underlying brain activity. Recordings *in vitro* and *in vivo* have shown that during (i.e., online to) rhythmic electromagnetic stimulation, neuronal spiking activity phase-aligns with the applied periodic electromagnetic force (Fröhlich and McCormick, 2010; Ozen et al., 2010; Ali et al., 2013; see also Reato et al., 2010 and review by Reato et al., 2013b). Converging evidence from human research confirms that, during stimulation, oscillatory brain activity as measured with electro-encephalography (EEG) and more recently with magneto-encephalography (MEG), phase-locks to rhythmic trains of stimulation (tACS: Helfrich et al., 2014b, Witkowski et al., 2015; rTMS: Thut et al., 2011b; Romei et al., 2015; see also Rosanova et al., 2009; Herring et al., 2015 for phase-locking to single pulse TMS). Hence, the electromagnetic force can be used to entrain, or synchronize, intrinsic brain oscillations (for reviews see: rTMS: Thut et al., 2011a; tACS: Herrmann et al., 2013; Fröhlich, 2015; otDCS: Marshall and Binder, 2013). Notably, such entrainment effects are more pronounced when the frequency of stimulation coincides with the dominant frequency of the stimulated neurons/areas (Ozen et al., 2010; Ali et al., 2013; Reato et al., 2013b; Schmidt et al., 2014; Romei et al., 2015), suggesting that frequency tuning constitutes a means for interacting with intrinsic neuronal network activity with some specificity. Besides these electrophysiological entrainment effects, corresponding changes in behavioral performance during stimulation have been reported. These consist of oscillatory patterns in performance measures imposed by rhythmic stimulation, or behavioral performance being biased in expected directions when the stimulation frequency matches known, task-related brain oscillations. For tACS/otDCS, such behavioral effects have been shown in various domains including perception (e.g., Feurra et al., 2011; Laczó et al., 2012; Neuling et al., 2012a; Brignani et al., 2013; Strüber et al., 2014; Helfrich et al., 2014a,b; Riecke et al., 2015), decision making (e.g., Sela et al., 2012), crossmodal integration (e.g., Cecere et al., 2015), motor control (e.g., Pogosyan et al., 2009; Joundi et al., 2012), and memory/cognition (e.g., Polanía et al., 2012; Santarnecchi et al., 2013; Vosskuhl et al., 2015). Analogous examples exist for rTMS (Romei et al., 2010, 2011, 2012; Chanes et al., 2013; Hanslmayr et al., 2014; Jaegle and Ro, 2014; Ruzzoli and Soto-Faraco, 2014; Quentin et al., 2015). This shows that tACS/otDCS and rTMS are viable tools to exert control over both brain oscillations and their associated functions. As a consequence, guiding online tACS/otDCS or rTMS by knowledge of task-related oscillatory brain activity may be a promising approach to enhance the specificity and effectivity of interventions.

Box 1 **Rhythmic transcranial brain stimulation techniques.**

*Rhythmic transcranial electrical stimulation* methods include *oscillatory transcranial Direct Current Stimulation (otDCS) and transcranial Alternating Current Stimulation (tACS)*, which both involve the application of a weak (typically ≤ 2 mA), periodically fluctuating electric current between two or more electrodes attached to the scalp. These techniques act on the stimulated tissue by inducing a subthreshold polarization, which does not trigger action potentials, but rather change the resting membrane potential and thus lead to a change in the firing rate or pattern of the stimulated neurons.

- otDCS involves a current that has either an anodal or cathodal polarity with respect to the target electrode, i.e., the direction of current flow is either directed inward (toward the electrode) or outward (away from that electrode), respectively. The current intensity oscillates at a specific frequency either between 0 and maximum amplitude (e.g., Marshall et al., 2006), or with an additional DC offset (e.g., Neuling et al., 2012a). Waveform shapes include boxcars, trapezoids, and sinusoids.
- In tACS, the current is typically sinusoidal, with the polarity of the current at each electrode reversing periodically between anodal to cathodal at a defined frequency, presumably leading to alternating hyper- and depolarization of neuronal membranes.

Stimulation waveforms can be in phase or in anti-phase across electrodes or electrode pairs. With montages of two electrodes, stimulation is always in anti-phase, i.e., when the current is positive under one electrode, it will be negative under the other. In montages with more than two electrodes, the montage can be set up in a way that the current waveforms are in phase, i.e., simultaneously either positive or negative between any given pair of electrodes. The relative phase between electrodes is thought to have either facilitatory or disrupting effect on coherence between areas (Polanía et al., 2012; Helfrich et al., 2014a).

*Transcranial Magnetic Stimulation in its repetitive (also called rhythmic) form (rTMS)* refers to the application of trains of magnetic pulses at specific frequencies. The TMS mechanism of action is qualitative different from tACS and otDCS. Magnetic stimulation exploits time-varying magnetic fields generated within a coil positioned on the scalp to induce electric currents in the brain. The rapidly changing induced electric field affects the transmembrane potentials and may lead to a depolarization of nerve cells, induce the generation of action potentials, and result in phase-reset of ongoing oscillations (Rosanova et al., 2009; Miniussi and Thut, 2010; Herring et al., 2015).

In addition to these well-documented online effects, prolonged stimulation with tACS/otDCS or rTMS often leads to offline changes in oscillatory brain activity that persist after the end of stimulation. However, oscillations that are entrained during rhythmic stimulation have been reported to remain stable only for maximally a few oscillatory cycles after stimulation offset (Marshall et al., 2006; Reato et al., 2013a, same data; Thut et al., 2011b; Hanslmayr et al., 2014). These short-lived post-tACS/rTMS reverberations that show phase-locking to the entraining field have also been termed “entrainment-echoes” (Hanslmayr et al., 2014). On the other hand, many of the offline effects on brain oscillations can be observed over much longer durations (for TMS, e.g., Thut and Pascual-Leone, 2010; for tACS, e.g., Zaehle et al., 2010), and may not simply reflect continuation of online entrainment. We refer here to these longer lasting offline effects as aftereffects.

One important open question is if and to what extent aftereffects on brain oscillations relate to entrainment effects observed during stimulation (e.g., Vossen et al., 2015). Given that entrainment dominates the online EEG recordings for stimulation at physiological frequencies (e.g., Thut et al., 2011b; Helfrich et al., 2014b; Romei et al., 2015), it is conceivable that some of these aftereffects are conveyed through direct interaction with brain oscillations that match the frequency of stimulation. This would imply that control over oscillatory brain activity during stimulation through entrainment transfers to some extent to the changes observed after stimulation. If this is the case, aftereffects should predominate in narrow frequency bands that match the stimulation frequency and its harmonics or subharmonics (Ali et al., 2013), or extend to other frequency bands through known physiological cross-frequency interactions (e.g., alpha-gamma, Spaak et al., 2012). Alternatively, aftereffects of tACS/otDCS and rTMS on brain oscillations may originate in other mechanisms unrelated to interaction with brain oscillations *per se*. In this case, stimulation would be expected to generate broadband modulations, or effects at frequencies that have no direct or indirect relationship with the stimulation frequency.

Here, we review EEG/MEG studies on changes in oscillatory brain activity after tACS/otDCS and rTMS. In light of the well-documented online entrainment effects that suggest direct and frequency-specific interactions with oscillatory brain activity, we ask to what extent a similar level of control over oscillatory activity and associated behavioral effects can be achieved beyond stimulation. To this end, we surveyed the literature for patterns of aftereffects in favor or against the hypothesis that direct interactions with oscillatory brain activity are at their origin. Because of the analogy between tACS/otDCS and rTMS in terms of evidence for online entrainment, we here considered studies that applied either type of stimulation, despite the differences in basic physiological mechanisms (see Box 1), and despite the rTMS field traditionally guiding the frequency of interventions not based on brain oscillations but on protocols known to induce either long term potentiation (LTP) and long-term depression (LTD) (e.g., Thickbroom, 2007).

We selected those tACS, otDCS, and rTMS studies that investigated EEG/MEG-aftereffects in healthy participants in terms of changes in power or connectivity (e.g., interhemispheric coherence) for at least 1 min following stimulation, i.e., when entrainment-echoes likely have ceased. In the following, we will refer to these lasting changes (regardless of the specific measure used) as aftereffects unless otherwise specified. For details please refer to the corresponding Table 1 (tACS/otDCS) and Table 2 (rTMS). Because of our focus on brain oscillations, we did not include studies that used other valuable measures to characterize aftereffects, such as motor evoked potential (MEP) (e.g., Moliadze et al., 2012) or behavioral outcomes (e.g., Laczó et al., 2012), but did not record EEG/MEG. According to the above criteria, we identified 22 tACS/otDCS studies (Table 1, total of 33 experiments) as well as 26 rTMS studies (Table 2, total of 33 experiments) investigating EEG/MEG-aftereffects. We asked three questions: How consistently are aftereffects on brain oscillations reported across studies? Do these aftereffects show replicable patterns across experiments that suggest direct interactions with brain oscillations as their origin (including effects on intrinsic activity at stimulation frequency and/or its (sub)harmonics, or through known cross-frequency interactions)? And to what extent do such aftereffects translate into predictable behavioral consequences?

**Table 1:** **Aftereffects of oscillatory transcranial direct current stimulation (otDCS) and transcranial alternating current stimulation (tACS) as assessed by EEG**.

**ID/study**	**Method**	**Stimulation frequency**	**Montage**	**Protocol**	**Effects at stimulation frequency**	**Effects in other frequency bands**	**Recording time**	**Duration of aftereffect**
1. Garside et al., 2015	tACS	δ 0.75	F3/F4 (5/5)	5 × 5' stimulation with 1-min intervals during sleep; 0.55 mA	Yes δ(broadband): suppressed power increase followed by rebound	No	Only 1 min intervals after each stimulation block assessed	At least 1 min
2. Eggert et al., 2013	otDCS	δ 0.75	F3/F4 (A) / ipsil. mastoids (4 × 0.8)	5 × 5′ with 1′ intervals during sleep; 0.26 mA	*Maybe (n.s.)* δ (broadband): power decrease	No	Only 1 min intervals after each stimulation block assessed	At least 1 min
3. Sahlem et al., 2015	otDCS	δ 0.75	F3/F4 (A) / ipsil. mastoids (4 × 1.1)	5 × 5′ with 1′ intervals during sleep; 0.6 mA	*Maybe (n.s.)* Slow δ: power increase	*Maybe (n.s.)* α: power increase	1 min intervals after each stimulation block and up to 60 min after stimulation	n.s.
4. Marshall et al., 2006	otDCS	δ 0.75	F3/F4 (A) / ipsil. mastoids (4 × 0.5)	5 × 5′ with 1′ intervals during sleep; 0.26 mA	Yes Slow and maybe broadband δ: power increase	Yes α: slow spindle activity increase	Max. 1 min after each stimulation block assessed	Only first 3 of 5 1 min interstimulation intervals
“(control exp.)	“	θ 5	“	“	No	Yes slow δ: power decrease; *Maybe (n.s.)* α: slow spindle activity decrease	Max. 1 min after each stimulation block assessed	Only first 3 of 5 1 min interstimulation intervals
5. Reato et al., 2013a (data from Marshall et al., 2006)	otDCS	δ 0.75	F3/F4 (A) / ipsil. mastoids (4 × 0.5)	5 × 5′ with 1′ intervals during sleep; 0.26 mA	Slow δ: slower decay rate of power/coherence	Not analyzed	4.5 h	Prolonged changes over the course of 4.5 h
6. Kirov et al., 2009 (Exp. 1)	otDCS	δ 0.75	F3/F4 (A) / ipsil. mastoids (4 × 0.5)	5 × 5′ with 1′ intervals during rest; 0.26 mA	Yes slow δ: power increase	Yes θ, β: power increase	1 min intervals after each stimulation block and after30/60 min	Only during 1 min intervals and immediately after stimulation but not 30 or 60 min later
“(Suppl. exp.)	“	“	“	5 x 5′ with 1′ intervals during task; 0.26 mA	Yes slow δ: power increase	Yes θ: power increase	1 min intervals after each stimulation block and after 30/60 min	Only during 1 min intervals and immediately after stimulation but not 30 or 60 min later
7. Antonenko et al., 2013	otDCS	δ 0.75	F3/F4 (A) / ipsil. mastoids (4 × 0.5)	6–8 × 4′ with >1′ intervals during sleep; 0.25 mA	Yes δ: power increase	Yes β: power decrease	Max. 1 min after each stimulation block assessed	δ: first 3 interstimulation intervals; β: only first interval
8. Vosskuhl et al., 2015	tACS	δ/θ below ITF	FPz/Pz (35/35)	3 × ~6′ during cognitive task; 0.4–1.3 mA	Yes θ: power increase	Not analyzed	within ~10 min after stimulation	At least up to 10 min
9. Pahor and Jaušovec, 2014	tACS	θ IAF-5	F3/Fp2 (35/70)	15′ during rest; 1–2.25 mA	Yes θ: ERD increase	Yes Lower α: power decrease; ERD increase	rest: within 5 min after tACS, task: 5–25 min after tACS	At least up to 25 min
“	“	“	P3/Fp2 (35/70)	“	Yes θ: power increase	Yes Lower α: power decrease; ERD increase	“	”
10. Marshall et al., 2011	otDCS	θ 5	F3/F4 (A) / ipsil. mastoids (4 × 0.5)	5 × 5′ with 1′ intervals during NREM sleep; 0.26 mA	No	Yes broadband δ/α power decrease	During 1 min intervals and immediately after stimulation, and after 30/60 min	At least 1 min but followed by rebound within 30 min, no difference after 60 min
“	“	“	“	5 × 5′ with 1′ intervals during REM sleep; 0.26 mA	No	Yes γ and maybe β power increase	Only 1 min intervals after each stimulation block assessed	At least 1 min
11. Vossen et al., 2015	tACS	α IAF	PO7/9//PO8/10 (35/35)	11–15′ (intermitten*t* 3 or 8 s on/off pattern) during task; 1.35–2 mA	Yes α: power increase	No	Within 2 min after stimulation	At least 2 min
12. Zaehle et al., 2010	tACS	α IAF	PO9/PO10 (35/35)	10′ during task; 0.8–3 mA	Yes α: power increase	Not analyzed	Within 3 min after stimulation	At least 3 min
13.Strüber et al., 2015	tACS	α IAF	Cz/Oz (35/35)	10′ (intermitten*t* 1 s on, /4–7 s off pattern) during task; 0.76 (± 0.30) mA	No	No	Only between intermittent tACS epochs	–
“	“	β/ γ 3.1^*^IAF	“	“	Not analyzed	No	“	–
14. Neuling et al., 2013	tACS	α IAF	Cz/Oz (35/35)	20' during task w/eyes open; ~1.5 mA	Yes α: power increase	Not analyzed	30 min	At least 30 min
“	“	“	“	20′ during task w/eyes closed; ~1.5 mA	Yes α: coherence increase	Not analyzed	“	“
15. Helfrich et al., 2014b	tACS	α 10	Cz/Oz (35/35)	20′ during task; 0.8–1 mA	Yes α: power increase	No	Within 3 min after stimulation	At least 3 min
16. Neuling et al., 2012a	otDCS	α 10	T7/T8 (A) (35/35)	2 × 21′ (on 2 days) during task; ~.45 mA AC modulation +1 mA DC offset	Yes α: power increase	Yes δ: power increase	Within 3 min after stimulation	At least 3 min
17. Wach et al., 2013	tACS	α 10	leftM1/right orbit (35/35)	10′ during rest; 1 mA	No	Yes γ: decrease in cortico-muscular coherence during isometric contraction)	38 min	At least 38 min
“	“	β 20	“	“	No	*Maybe (n.s.)* γ: power: decrease during isometric contraction	“	n.s.
18. Krause et al., 2013	tACS	β 20	leftM1/right orbit (35/35)	15′ during rest; 1 mA	No	Not analyzed	Between 5 and 13 min after stimulation	–
19. Strüber et al., 2014 (Exp. 1)	tACS	γ 40	P7-PO7/P8-PO8 (35/35)	15′ during task, anti-phase; 1.02 ± 0.62) mA	Yes γ : increase of interhemispheric coherence	Not analyzed	Within 3 min after stimulation	At least 3 min
“ (Exp. 2)	“	θ 6	“	15′ during task, anti-phase; 0.50 (±0.19) mA	No	No	”	–
“ (Exp.3)	“	γ 40	C3/C4//O1/O3 (4 × 15.21)	15′ during task, in-phase; 1.23 (±0.35)	No	No	~39 min	–
“ (Exp. 3)	“	θ 6	“	15′ during task, in-phase; 1.40 (±0.40) mA	No	No	”	–
20. Helfrich et al., 2014a	tACS	γ 40	10 el. bilaterally over posterior cortex (10 × 1.1)	20′ in-phase or anti-phase during task; 1 mA	Yes γ: Increase in broadband interhemispheric coherence after in-phase vs. anti-phase stimulation	No	~32 min	Max. 20 min
21. Chaieb et al., 2014	tACS	5000	Left M1/right orbit (16/45)	10′ during rest; 1 mA	Not analyzed	Yes δ: power decrease	14 min	Between 2 and less than 7 min
22. Antal et al., 2008	tACS	1, 10, 45	Left M1/right orbit (16/50)	5′ per freq. w/ >20 min break during rest; 0.4 mA	No	No	14 min	–
“	otDCS	5, 10, 15	Left M1/right orbit; both A and C (16/50)	4′ per freq. w/ >20 min break during rest; 0.25 mA	No	No	4 min	–

*Studies are sorted from top to bottom first in ascending order by stimulation frequency and then electrode montage (from anterior to posterior brain), except when a study reports experiments using different frequencies, in which case the separate experiments are grouped under the stimulation frequency of the main experiment. Aftereffects are expressed in terms of effects at stimulation frequency, other frequencies and duration. IAF, Individual alpha frequency; ITF, Individual theta frequency; n.s., not significant. Stimulation intensity is indicated as maximum current (in mA). Note that typically, intensity refers to maximum amplitude in otDCS studies and to peak-to-peak amplitude in tACS studies. The numbers in parenthesis in the “montage” columns refer to electrode size*.

**Table 2:** **Aftereffects of repetitive (also called rhythmic) transcranial magnetic stimulation (rTMS) as assessed by EEG/MEG**.

**ID/study**	**Method**	**Stimulation frequency**	**Target**	**Protocol**	**Effects at stimulation frequency**	**Effects in other frequency bands**	**Recording time**	**Duration of aftereffects**
1. Schutter et al., 2001	Single frequency	δ 1 Hz	DLPC	1 × 20 min train; 2000 p; 130% MT	Not analyzed	Yes θ: power increase	65 min	65 min
2. Wozniak-Kwasniewska et al., 2013	Single frequency	δ 1 Hz	DLPC	4 trains × 3.33 min ITI 33 s; 800 p; 120% rMT	Yes δ: power decrease	Yes θ: power decrease β,γ: power in-& decrease	10 min	10 min
”	Single frequency	α 10 Hz	DLPC	10 Hz 16 × 5 s train ITI 54 s; 800 p; 120% rMT	No	Yes δ, θ: power decrease β,γ: power in-& decrease		
”	iTBS	γ/θ 50 Hz@5 Hz	DLPC	6 × 2 s trains × 4 ITI 229 s; 792 p; 80% rMT	Yes γ: power in-&decrease θ: power decrease	Yes δ: power decrease; β: power in-& decrease		
”	cTBS	γ/θ 50 Hz@5 Hz	DLPC	4 × 17 s trains ITI 280 s; 792 p; 80% rMT	Yes γ: power in-&decrease θ: power decrease	Yes δ: power decrease β: power in-& decrease		
3. Chen et al., 2003	Single frequency	δ 0.9 Hz	PM	1 × 15 min train; 818 p; 90% aMT	Not analyzed	Yes α, β: task-related power decrease α: motor areas coherence increase	30 min	15 min
4. Strens et al., 2002	Single frequency	δ 1 Hz	M1	1 × 25 min train; 1500 p; 90% aMT	Not analyzed	Yes α: intra and inter- hemispheric coherence increase (rest, task)	50 min	25 min intra-hemispheric coh, few minutes inter-hemispheric coh
5.Tamura et al., 2005	Single frequency	δ 1 Hz	M1	1 × 10 min train; 600 p; 95% rMT	Not analyzed	Yes β: reduction of movement-related rebound	10 min (starting 10 min after stimulation)	10 min
6.Capotosto et al., 2014	Single frequency	δ 1 Hz	l/rAG; l/r FEF; l/r IPS	1 × 1 min train; 60 p; rMT	Not analyzed	Yes α (IAF-2): power increase (AG), intra-hemispheric coherence increase (rAG)	2 min	2 min
7.Thut et al., 2003	Single frequency	δ 1 Hz	V1/V2	1 × 10 min train; 600 p; 110% PT	Not analyzed	Yes α: reduction of visual-induced desynchronization	8 min	8 min
8.Serrien et al., 2002	Single frequency	θ 5 Hz	SMA	1 × 10 s train; 50 p; 90% rMT	No	Yes α, β: interhemispheric coherence decrease between MIs	25 min	Few min after stimulation
9. Oliviero et al., 2003	Single frequency	θ 5 Hz	M1	1 × 10 s train; 50 p; aMT	Not analyzed	Yes α (10.7–13.6 Hz): intra-hemispheric coherence decrease (task)	50 min	Few min after the end of stimulation
10. Huber et al., 2007	Single frequency	θ 5 Hz	M1	5 × 6 × 10 s trains ITI 5 s; 1500 p; 90% rMT	No	Yes SWA (1-4.5 Hz): power increase (sleep)	First NREM sleep cycle (60 min)	30 min
11. Jing and Takigawa, 2000	Single frequency	α 10 Hz	DLPC	2 × 3 s trains ITI 5 min; 60 p; rMT	Yes α: direct coherence increase	No	5 min	5 min (not studied longer)
12. Okamura et al., 2001	Single frequency	α 10 Hz	DLPC	2 × 3 s trains ITI 300 s; 60 p; rMT	Yes α: change in peak frequency and power	Yes δ,θ, β,γ: change in peak frequency and power	5 min	5 min
13. Griskova et al., 2007	Single frequency	α 10 Hz	DLPC	100 × 2 s trains ITI 10 s; 2000 p; 110% rMT; 90% rMT sham	No	Yes δ: power increase	10 min	10 min
14. Weisz et al., 2014	Single frequency	α IAF	Auditory cortex	20 × ± 5 s trains; 100 p; 50% MSO	No	No	5 min	NA
15. Graf et al., 2001	Single frequency	β 20 Hz	DLPC	40 × 2 s trains ITI 28 s; 1600 p; 90% rMT	No (waking or sleep EEG)	No (waking or sleep EEG)	10 min (waking EEG); 8 h	NA
16. Veniero et al., 2011	Single frequency	β 20 Hz	M1	10 × 0.45 s trains ITI 14.55 ms; 400 p; rMT	No	Yes α: power increase (ERS)	10 min	5 min
17. Schutter et al., 2003	Single frequency	β 25 Hz	Cerebellum	80 × 10 s trains ITI 5 s; 2000 p; MT	No	Yes γ: change in prefrontal asymmetry	15 min	15 min
18. Grossheinrich et al., 2009	cTBS	γ/θ 50 Hz@5 Hz	DLPC	1 x 40 s train; 600 p; 80% rMT	No γ not analyzed	No	50 min	NA
”	cTBS	γ/θ 50 Hz@5 Hz	mPFC	1 × 40 s train; 600 p; 80% rMT	No γ not analyzed	No		NA
”	iTBS	γ/θ 50 Hz@5 Hz	DLPC	20 × 2 s trains ITI 8 s; 600 p; 80% rMT	No γ not analyzed	Yes α: power increase		50 min (not studied longer)
”	iTBS	γ/θ 50 Hz@5 Hz	mPFC	20 × 2 s trains ITI 8 s; 600 p; 80% rMT	No γ not analyzed	No		NA
19. McAllister et al., 2011	cTBS	γ/θ 50 Hz@5 Hz	M1	1 × 40 s train; 600 p, 80%aMT	No γ not analyzed	No	8 min	NA
20. Noh et al., 2012	cTBS	γ/θ 50 Hz@5 Hz	M1	1 × 20 s train; 300 p; 80% aMT	Yes θ: power increase (ERS). γ not analyzed	Yes μ (10–12.5 Hz), β: power increase (ERS)	30 min	30 min
21. Vernet et al., 2013	cTBS	γ/θ 50 Hz@4.7 Hz	M1	1 × 40 s train; 600 p; 80% aMT	Yes θ: TMS-induced synchronization decrease; power decrease (rest) γ not analyzed	Yes α: power decrease (ERD); β: TMS-induced synchronization increase (ERS) β: power decrease (rest)	40 min	40 min
22. Shafi et al., 2014	cTBS	γ/θ 50 Hz@5 Hz	M1	1 × 40 s train;600 p; 80% aMT	Yes θ: clustering coefficient increase γ not analyzed	Yes α: connectivity decrease high-β: connectivity increase	10 min	10 min
23. Assenza et al., 2015	iTBS	γ/θ 50 Hz@5 Hz	M1	20 × 2 s trains ITI 8 s; 600 p; 80% rMT	No γ NA	Yes δ: power increase	30 min	5 min
24. Rizk et al., 2013	cTBS	γ/θ 50 Hz@6 Hz	rPPC	1 × 33 s train; 801 p; 90% rMT	Yes 30 Hz coherence decrease	Yes α: coherence decrease (rPPC-rest of the brain)/ increase (lPPC-rest of the brain)	40 min	30–40 min
”	cTBS	γ/θ 50 Hz@6 Hz	rFEF		No	No		NA
25. Marshall et al., 2015	cTBS	γ/θ 50 Hz@5 Hz	R/L FEF	1 × 40 s train; 600 p; 80% aMT	Yes γ: increased stimulus induced synchronization	Yes α: reduced stimulus induced desynchronization	30 min	30 min
26. Schindler et al., 2008	cTBS	γ/α 30 Hz@10 Hz	rFEF	1 × 33 s train; 600 p; 80% rMT	Yes γ/α: synchronization increase	Yes δ, θ, β: synchronization increase (compared to unstimulated hemisphere), absolute decrease	60 min	60 min

*Studies are order from top to bottom according to stimulation frequency and target site (from anterior to posterior brain). Aftereffects are expressed in terms of effects at stimulation frequency, other frequencies and duration. DLPFC, dorsolateral prefrontal cortex; mPFC, medial prefrontal cortex; PM, premotor cortex; M1, primary motor cortex; rAG, right angular gyrus; FEF, frontal eye field; IPS, intraparietal sulcus; PPC, posterior parietal cortex; SMA, supplementary motor area; V1, V2, early visual cortex; MT, motor threshold; r/aMT, resting/active motor threshold; MSO, maximum stimulator output; PT, phosphene threshold; ITI, intertrain interval; p, pulses; NA, not applicable*.

## How consistently are aftereffects on brain oscillations reported?

### tACS/otDCS studies

Oscillatory transcranial electrical stimulation is often accompanied by aftereffects on neural synchrony (Table 1). Of the 22 tACS/otDCS studies included, only three did not report any significant aftereffects (Antal et al., 2008; Krause et al., 2013 in their healthy control group; Strüber et al., 2015). The respective null effects might be explained by insufficient stimulation intensity (Antal et al., 2008), population differences between Parkinson's disease patients and healthy control participants (Krause et al., 2013), or insufficient stimulation duration (Strüber et al., 2015). All other studies report at least one change in one measure of oscillatory activity (power/coherence, or relative changes thereof).

In terms of duration of tACS/otDCS aftereffects, only few data are available as analyses were mostly restricted to short stimulation-free intervals between successive blocks of stimulation (Marshall et al., 2006; Antonenko et al., 2013; Eggert et al., 2013; Garside et al., 2015), and/or to maximally 3 min after stimulation (Zaehle et al., 2010; Neuling et al., 2012a; Strüber et al., 2014; Helfrich et al., 2014b; Vossen et al., 2015). In those studies that looked at slightly longer intervals, durations of aftereffects are variable, ranging between a few minutes (Chaieb et al., 2014) up to about half an hour (Kirov et al., 2009; Neuling et al., 2013; Wach et al., 2013; Helfrich et al., 2014a), but appear to subside by 60 min after stimulation at the latest (Kirov et al., 2009; Marshall et al., 2011; Sahlem et al., 2015), although in one exception changes only manifested over the course of several hours (Reato et al., 2013a). Notably, aftereffects have also been found to rebound, i.e., an initial power suppression may turn into a power enhancement (Marshall et al., 2011). In addition, analyses of inter-stimulation periods during otDCS revealed that changes observed early into stimulation may wear off before the end of total stimulation time (Marshall et al., 2006; Kirov et al., 2009; Antonenko et al., 2013) or even reverse (Garside et al., 2015), suggesting that homeostatic mechanisms might actively work against aftereffects.

### rTMS-studies

For rTMS, aftereffects on brain oscillations are frequently reported as well (Table 2), as only four out of the 26 studies did not report aftereffects (Graf et al., 2001; Grossheinrich et al., 2009 in three out of four experiments; McAllister et al., 2011; Weisz et al., 2014).

Similar to tACS/otDCS, evaluating the duration of the rTMS aftereffects is difficult as the 22 studies reporting significant aftereffects covered different time windows, ranging from a few minutes (Capotosto et al., 2014) to 65 min after stimulation (Schutter et al., 2001), with only eight studies recording brain activity up to recovery (Serrien et al., 2002; Strens et al., 2002; Chen et al., 2003; Oliviero et al., 2003; Huber et al., 2007; Veniero et al., 2011; Assenza et al., 2015; Marshall et al., 2015). Longer recording windows have been mostly considered for theta burst stimulation (TBS) protocols (Schindler et al., 2008; Grossheinrich et al., 2009; Noh et al., 2012; Rizk et al., 2013; Vernet et al., 2013) which consistently showed that the aftereffects can outlast the stimulation for 40–60 min. For the rTMS protocols using single frequencies, the results are more varied, with experiments using low frequency rTMS (0.9–1 Hz) reporting aftereffects lasting 15–65 min (Schutter et al., 2001; Strens et al., 2002; Chen et al., 2003) and experiments testing higher frequencies (5–25 Hz) reporting aftereffects of 5–30 min duration (Huber et al., 2007; Veniero et al., 2011). Note that the latter studies varied considerably in number of delivered pulses and intensity of stimulation, whereas TBS studies followed more strictly a standard protocol (usually 50 Hz@5 Hz, 600 pulses, 80% aMT, but see Wozniak-Kwasniewska et al., 2013).

In summary, most of the reviewed studies report tACS/otDCS- and rTMS-aftereffects on brain oscillations. In the following section, we examine whether consistent patterns in these aftereffects can be identified across studies, with an emphasis on those patterns that would be suggestive of direct interaction with intrinsic oscillations (entrainment) as their origin.

## Do aftereffects on oscillatory brain activity show replicable patterns across studies?

As an index of control over brain oscillations, we assessed whether a consistent relationship exists between the externally applied stimulation frequency and affected frequencies in intrinsic oscillatory activity. Specifically, we surveyed the literature for evidence that stimulation frequency can predict the observed changes in intrinsic frequencies (i.e., effect frequencies), and vice versa, whether effect frequencies are preferentially associated with specific stimulation frequencies. Patterns that would indicate that the aftereffects primarily originate from direct interactions with oscillatory brain activity are (i) a match between stimulation and effect frequencies (Thut et al., 2011b; Schmidt et al., 2014; Helfrich et al., 2014a,b), (ii) effects at (sub)harmonics (Ali et al., 2013), or (iii) specific cross-frequency changes that have been related to intrinsic network coupling modes in electrophysiological recordings (for review see e.g., Engel et al., 2013), such as amplitude-amplitude coupling (e.g., gamma-alpha, Spaak et al., 2012) or phase-amplitude coupling (e.g., theta-gamma, Canolty et al., 2006), which would point to network effects.

### tACS/otDCS-studies

**Figure 1:**
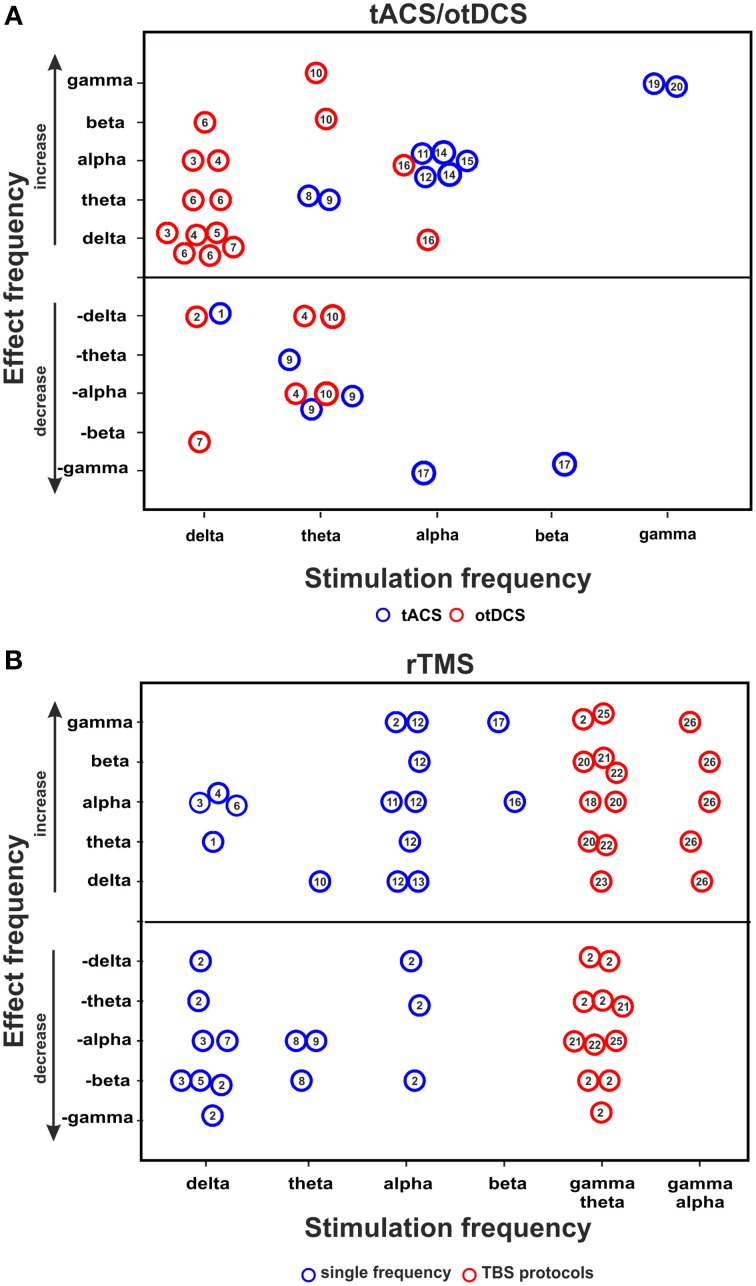
**tACS/otDCS and rTMS aftereffects on brain oscillation**. Relationship between stimulation frequency and effect frequency as inferred from **(A)** combined tACS/otDCS-EEG/MEG studies, and **(B)** combined rTMS-EEG/MEG studies. The figure collapses across EEG measures used (power or coherence). Each circle represents a reported effect (refer to data in Tables 1, 2 for tACS/otDCS and rTMS, respectively). Numbers in circles correspond to the respective study ID in the leftmost column in the relevant table. Circles above the horizontal line indicate a power/coherence increase, circles below the line indicate a power/coherence decrease. Null effects are not included; consequently this figure over-represents positive results.

Figure 1A summarizes the relationship between stimulation- and effect frequencies for tACS/otDCS (illustrating the data of Table 1, 5 kHz-tACS not included). The largest body of research has focused on the aftereffect of *slow frequency (0.75 Hz) stimulation over frontal areas* (also called transcranial slow oscillation stimulation/tSOS) mostly during sleep (Marshall et al., 2006, 2011; Antonenko et al., 2013; Eggert et al., 2013; Reato et al., 2013a; Garside et al., 2015; Sahlem et al., 2015; see also Table 1, rows 1–9). Using similar experimental protocols (anodal stimulation over F3/F4 at 0.75 Hz), these studies showed that delta-otDCS/tACS leads to somewhat reliable and replicable EEG effects in the delta band, often alongside changes in other frequency bands (see Figure 1A, “effect frequency” in “stimulation frequency” delta bin). Conversely, effects in the delta band were rarely observed with other stimulation frequencies (Marshall et al., 2006 [5 Hz]; Marshall et al., 2011 [5 Hz]; Neuling et al., 2012a [individual alpha frequency/IAF]; Chaieb et al., 2014 [5 kHz]) (see Figure 1A, “stimulation frequency” in “effect frequency” delta bin). This points to a reasonable match between stimulation and effect frequency for slow frequency stimulation and suggests a direct interaction of frequency-tuned tACS/otDCS with intrinsic brain activity through resonance of a slow oscillating network. However, mixed results have been reported regarding the direction of modulation (i.e., enhancement: Marshall et al., 2006; Kirov et al., 2009; Antonenko et al., 2013; Sahlem et al., 2015; vs. suppression: Eggert et al., 2013; Garside et al., 2015), and regarding accompanying spectral changes (e.g., alpha increase after otDCS during sleep: Marshall et al., 2006; vs. theta increase during wakefulness: Kirov et al., 2009). This has been suggested to reflect dependence of the response on population characteristics (Eggert et al., 2013), on whether both hemispheres are stimulated in-phase or in anti-phase (see Box 1 for definition; Garside et al., 2015), as well as on brain state (Marshall et al., 2006; Kirov et al., 2009).

Another series of studies investigated the aftereffects of *posterior alpha tACS* on spectral measures (Zaehle et al., 2010; Neuling et al., 2012a, 2013; Helfrich et al., 2014b; Vossen et al., 2015; see Table 1, rows 15–21). This reliably resulted in alpha power increases (see Figure 1A), again suggesting a good match between stimulation- and effect frequency. However, sometimes alpha-activity has also been found to increase after frontal slow delta stimulation (Figure 1A; Marshall et al., 2006; Sahlem et al., 2015), and to decrease after theta stimulation (Figure 1A; Marshall et al., 2006, 2011; Pahor and Jaušovec, 2014). Some alpha changes may thus reflect other mechanisms than specific oscillatory interactions (see also rTMS section below and Veniero et al., 2011).

*Theta tACS/otDCS* has rarely been followed by lasting changes in theta activity (Pahor and Jaušovec, 2014; Vosskuhl et al., 2015). Rather, a variety of responses in other frequency bands is observed (Marshall et al., 2006, 2011; Pahor and Jaušovec, 2014), without any obvious consistent pattern (Figure 1A). Theta changes have instead been observed after frontal slow frequency stimulation in awake participants (Marshall et al., 2011).

Finally, recent *posterior gamma tACS* studies (Helfrich et al., 2014a; Strüber et al., 2014) found that 40 Hz (but not 6 Hz) tACS either enhanced or weakened interhemispheric coherence in the gamma range depending on whether bilateral occipital stimulation is delivered in-phase or in anti-phase, again suggesting some match between stimulation- and effect frequency, although with a discrepancy between the narrow frequency bin of stimulation (40 Hz) and the rather broadband gamma effect (Helfrich et al., 2014a; Strüber et al., 2014).

With respect to cross-frequency tACS/otDCS-aftereffects that may be indicative of targeted interventions into oscillatory network activity, some studies reported that delta-otDCS during sleep enhanced alpha spindles (Marshall et al., 2006; Sahlem et al., 2015). Because these spindles are thought to be driven by slow oscillations during slow wave sleep (Steriade, 2006; Mölle et al., 2011), this may constitute supporting evidence for otDCS interactions with oscillatory network activity. However, other studies with similar stimulation parameters but arguably different state variables failed to observe this effect (Kirov et al., 2009; Antonenko et al., 2013; Eggert et al., 2013). Recently, Helfrich et al. (2014a) observed reduced alpha-power during stimulation with 40 Hz (gamma-)tACS, in line with the known inverse gamma-alpha relationship (e.g., Fries et al., 2001; Spaak et al., 2012). However, this interaction did not lead to stable alpha aftereffects.

In summary, the collective evidence for frequency-specificity of otDCS/tACS aftereffects is mixed at best. Although aftereffects at the stimulation frequency or a physiologically related frequency band have been observed after stimulation at delta, alpha, and gamma frequencies, experimental conditions and outcomes have overall been too variable, and identically performed replications too few, to unequivocally establish either direct frequency-matched modulation originating in entrainment, or cross-frequency phenomena which would indicate interactions through intrinsic coupling modes. In addition, it needs to be pointed out that the coverage of frequency bands is incomplete in several studies (especially for alpha stimulation) (see Table 1; “effects in other frequency bands”), with a considerable number of studies focusing on selective frequency bands. This may skew the conclusions favorably toward frequency specificity.

### rTMS-studies

Figure 1B summarizes the relationship between stimulation and effect frequencies across rTMS studies (illustrating the data of Table 2). With one exception (Weisz et al., 2014), none of these studies specifically tested the idea that rTMS can interact with brain oscillations in a controlled manner, but investigated electrophysiological aftereffects of rTMS in light of evidence that patterned transcranial magnetic stimulation can lead to long term potentiation/depression (LTP/LTD)-like phenomena (e.g., Thickbroom, 2007). Accordingly, many papers often refer to the classical distinction between excitatory and inhibitory rTMS (low vs. high frequency rTMS, continuous TBS vs. intermittent TBS) rather than to canonical frequency bands. As a result, not all papers did consider EEG effects at stimulation frequency. Collapsing the results across all studies (see Figure 1B) reveals that rTMS at one frequency typically leads to broadband aftereffects spanning more than one frequency band, as summarized below.

rTMS at *delta and theta* frequencies (0.9–1 vs. 5 Hz) has been reported to generally induce broadband EEG power modulations, with the exception of rTMS over motor areas which seems to affect activity in a more restricted (alpha/beta) frequency band (Serrien et al., 2002; Strens et al., 2002; Chen et al., 2003; Oliviero et al., 2003; Tamura et al., 2005). The only study investigating changes in the delta band after delta-rTMS reported a power decrease (Wozniak-Kwasniewska et al., 2013).

Conflicting results have been reported for rTMS at alpha frequency, i.e., *alpha-rTMS*. Some studies described increases in alpha power or connectivity (Jing and Takigawa, 2000; Okamura et al., 2001), although not exclusively so (Okamura et al., 2001). Others did not find any aftereffect in the alpha band (Griskova et al., 2007; Weisz et al., 2014). Alpha power increase has also been reported after *beta-rTMS* (Veniero et al., 2011), which has led to the suggestion that alpha-band increases may be a generic response of cortical areas cycling at alpha frequency independent of stimulation protocol (Veniero et al., 2011).

Several studies using continuous theta-burst stimulation (cTBS), consisting of *gamma bursts applied at theta frequency*, reported aftereffects in the theta-band on both connectivity (Shafi et al., 2014) and power (Noh et al., 2012; Vernet et al., 2013; Wozniak-Kwasniewska et al., 2013), albeit in opposite directions. When aftereffects were probed for changes in the gamma band (*n* = 4 studies) a complex pattern of gamma power and connectivity modulation was observed (Schindler et al., 2008; Rizk et al., 2013; Vernet et al., 2013; Marshall et al., 2015). Yet, aftereffects of cTBS were not restricted to the theta and gamma bands, and effects in these frequency bands were also reported across many other studies using other frequencies of stimulation. A similarly inconsistent picture emerges for intermittent theta-burst stimulation (iTBS), for which decreases in gamma/theta power have been reported in one out of three papers (Wozniak-Kwasniewska et al., 2013) alongside increases in alpha (Grossheinrich et al., 2009), beta (Wozniak-Kwasniewska et al., 2013) and delta power (Wozniak-Kwasniewska et al., 2013; Assenza et al., 2015).

In summary, many rTMS-studies did not analyse the aftereffects at stimulation frequency. Of those that did, a considerable fraction did not report any effects in this frequency band (Graf et al., 2001; Serrien et al., 2002; Schutter et al., 2003; Griskova et al., 2007; Huber et al., 2007; Grossheinrich et al., 2009; McAllister et al., 2011; Veniero et al., 2011; Weisz et al., 2014; Assenza et al., 2015). Whilst not providing much evidence for targeted interactions with brain oscillations *per se*, these results are relevant for two reasons. They suggest that rTMS, and possibly NIBS in general (see also discussion), is likely to lead to aftereffects in brain oscillations that are largely frequency-unspecific, and by extension, represent confounds for those attempting to control brain oscillations and associated functions through direct interaction via externally applied stimulation.

## Are EEG aftereffects accompanied by behavioral changes, in line with known correlative EEG-behavioral links?

The most promising evidence for behavioral aftereffects that correspond to the reported tACS/otDCS-induced EEG modulations has been provided by those studies using frontal 0.75 Hz tACS/otDCS aiming to either enhance or disrupt slow waves during sleep, a rhythm that has been associated with memory consolidation (e.g., Rasch and Born, 2013). As expected based on the known correlation between EEG slow wave activity and memory, several of these studies reported changes in declarative memory performance after frontal slow wave tACS/otDCS (Marshall et al., 2006, 2011; Kirov et al., 2009; Antonenko et al., 2013; Garside et al., 2015), while others failed to find an effect (Eggert et al., 2013; Sahlem et al., 2015). Of note, Garside et al. (2015) reported a negative correlation between the individual changes in slow/delta power and the performance on a verbal learning task (in the absence of a group effect on task performance), although it has to be pointed out that their sample was too small (*n* = 7) to allow strong conclusions. Collectively, these findings are promising but given that the neural and behavioral measures were generally obtained up to several hours apart, it is debatable whether the behavioral changes reflect a neural aftereffect or rather the interaction with an ongoing process in the acute phase of the experiment.

Behavioral effects were also studied using gamma-tACS (40 Hz) (Helfrich et al., 2014a; Strüber et al., 2014). These studies aimed to induce an oscillatory signature that has been associated in previous EEG studies with the prevalence of a specific apparent motion percept (Helfrich et al., 2014a; Strüber et al., 2014). Neither Strüber et al. (2014), nor Helfrich et al. (2014a) found aftereffects on perception, despite an online modulation of interhemispheric connectivity in the latter study. Yet, it has to be noted that Helfrich et al. (2014a) assessed the perceptual aftereffects long after the EEG modulation had subsided.

In studies on the cognitive impact of theta-tACS, short term memory performance did not improve beyond stimulation despite an online effect on memory (Vosskuhl et al., 2015), whereas Pahor and Jaušovec (2014) reported lasting improvement on tasks presumed to assess fluid intelligence for up to 25 min that coincided with a decrease in alpha activity. All other reviewed studies either did not assess whether the observed EEG/MEG aftereffects were correlated with meaningful behavioral consequences (Zaehle et al., 2010; Neuling et al., 2012a; Wach et al., 2013; Chaieb et al., 2014; Vossen et al., 2015), or found no such behavioral changes (Neuling et al., 2013).

With regard to rTMS, only few studies assessed its effects on cognitive tasks in relation to the outcome on brain oscillations. Marshall et al. (2015) showed that gamma/theta cTBS over frontal eye fields can modulate alpha and gamma power over the parieto-occipital cortex and concurrently impair performance in a cued spatial attention task for 16 min. Applying the same stimulation protocol, Rizk et al. (2013) also showed that posterior parietal cortex stimulation can induce neglect-like behavior during a visual exploration task lasting at least 15 min (not tested longer). This was however associated with a change of alpha connectivity between stimulated cortex and its homologous area and between both parietal areas and the rest of the brain, not with any effect at the stimulation frequencies. Interestingly, the behavioral effect of the stimulation was dependent on individual alpha band connectivity strength prior to stimulation.

In summary, only few studies looked at both behavioral and oscillatory aftereffects simultaneously. In those that did, many did not observe lasting behavioral changes. Only one study (Garside et al., 2015) reports a direct correlation between individual neural and behavioral aftereffects. Hence, at present, there is too little information about the behavioral impact of rhythmic stimulation-induced aftereffects to draw firm conclusions.

## Discussion

Our review suggests that aftereffects on oscillatory brain activity are common after otDCS, tACS, and rTMS, yet do not reveal a consistent pattern across studies relative to frequency of stimulation. For tACS/otDCS, only a fraction of studies have reported aftereffects that would be expected if they were related to entrainment of frequency-matched brain oscillations during stimulation, such as corresponding (cross-)frequency changes in EEG/MEG and associated behavior. For rTMS, the reported EEG/MEG aftereffects are broadband throughout, and range across multiple canonical frequency bands. Collectively, these data illustrate that despite some promising reports of controlled interventions into brain oscillations beyond stimulation, the overall evidence so far is weak. Below, we discuss some of the challenges that need to be overcome in order to test whether such interventions are viable. We suggest ways to optimize existing stimulation protocols, and outline future research directions to close the gaps in the existing evidence.

### Competing mechanisms of action of NIBS techniques

The most promising attempts to induce aftereffects in directly targeted brain oscillations and their associated functions have been made in tACS/otDCS studies. However, collectively, the tACS/otDCS results are mixed, with a considerable number of studies reporting effects that are independent of stimulation frequency. This illustrates that neural oscillations are responsive to electrical stimulation in general, in line with frequently reported changes in oscillatory brain activity after NIBS with other non-rhythmic protocols such as transcranial direct current stimulation (e.g., Lapenta et al., 2013; Puanhvuan et al., 2013; Spitoni et al., 2013; Hsu et al., 2014) or even static magnetic stimulation (Gonzalez-Rosa et al., 2015). Hence, frequency-tuned modulation of oscillatory brain activity will likely be accompanied by effects on brain oscillations that originate from mechanisms other than entrainment (e.g., forms of plasticity). In order to enhance control over aftereffects, the relative contribution of these different (possibly competing) mechanisms of action needs to be investigated. In addition, it remains to be established whether stimulation parameters can be optimized to favor one mechanism over another.

The idea that entrainment, or resonance, of cortical networks cannot explain all aftereffects on brain oscillations is in line with recent data from our group (Vossen et al., 2015). We found that tACS-induced aftereffects around stimulation frequency were observed even when the optimal condition for entrainment (i.e., continuous stimulation with a periodic field at the intrinsic frequency) was explicitly disrupted, and despite the absence of lasting phase alignment after tACS offset. One candidate mechanism that could lead to lasting aftereffects independently of entrainment are LTP- or LTD-like processes akin to those suggested to operate during non-rhythmic NIBS, such as tDCS (Nitsche et al., 2008, 2009; Ziemann and Siebner, 2008; Fritsch et al., 2010). Notably however, there is evidence from tACS-studies that online entrainment and aftereffects on brain oscillations may not be entirely unrelated (Helfrich et al., 2014a,b). Helfrich et al. (2014a,b) found the strength of entrained oscillatory power to be positively correlated with the strength of the aftereffects across participants. This important result (reproduced across two experiments using different stimulation frequencies, Helfrich et al., 2014a,b) suggests that entrainment effects online to tACS may influence the aftereffects, and hence may provide an opportunity for targeted intervention lasting beyond stimulation. Importantly also, Helfrich et al. (2014b) showed that these online and offline effects are of different quality, with the online effects observed at a narrow frequency peak, and the offline effects seen in a broader band around this peak discounting the trivial explanation that the correlated online and offline effects may simply reflect the same phenomenon measured at two time points. In line with these considerations, some accounts propose that entrainment of brain oscillations may specifically strengthen (or weaken) the targeted neuronal circuits by spike-timing dependent plasticity (STDP), which constitutes a hybrid account between immediate entrainment and longer lasting LTP/LTD-mechanisms (see Zaehle et al., 2010, for a computational model; Vossen et al., 2015 for a conceptual extension). The general idea is that small timing differences between tACS-imposed (entrained) and intrinsic cortical rhythms may lead to strengthening or weakening of the corresponding oscillatory neuronal circuit by modifying the efficacy of its component synapses. More specifically, the tACS-induced periodic hyper- and depolarization of neural membranes could impose a temporal window that modulates the spiking activity of stimulated neurons by raising or lowering the probability of an action potential as a function of phase in the stimulation cycle (as compared to a direct induction of action potentials). This would result overall in greater regularity in firing patterns and allow the modification of specific recurrent loops, involving feedback from relay neurons. As the stimulated neurons are more likely to fire at a specific point in the stimulation cycle, only feedback potentials that arrive in close temporal proximity to this time point (i.e., with a period slightly longer or shorter than the stimulation period) will result in synaptic strengthening/LTP or weakening/LTD, respectively.

Please note that so far, the only evidence available for this idea stems from a computational model (Zaehle et al., 2010). Experiments on cell cultures and *in-vivo* animal studies provide no insight as none of them have observed effects that last beyond stimulation offset (reviewed in Reato et al., 2013b; see also Strüber et al., 2015, for a discussion). The STDP account is only one of several possible models, some of which include more complex biologically plausible modeling (Fröhlich, 2015). Future studies will need to provide further insight into how and to what extent online entrainment contributes to offline effects at the neuronal level if we want to capitalize on frequency-tuned interventions that modulate specific oscillatory networks also beyond the duration of stimulation.

### rTMS vs. tACS/otDCS

Compared to the tACS/otDCS-literature, the evidence for a controlled lasting modulation of brain oscillations is particularly thin for rTMS, with aftereffects ranging across several frequency bands without any evidence for frequency specificity. This discrepancy may be explained by the difference in the physiological mechanisms by which tACS/otACS and rTMS affect neuronal activity: whereas tACS/otACS modulates membrane potentials without discharging neurons (Paulus, 2011), TMS elicits action potentials (Barker et al., 1987). Moreover, the stimulation techniques also differ in terms of spatial resolution, with tACS/otDCS affecting a significantly larger neural population than rTMS (e.g., Sparing and Mottaghy, 2008). Accordingly, it is conceivable that electrical and magnetic stimulation induce fundamentally different long-term plasticity effect. Nonetheless, rTMS has been shown to cause online entrainment (Thut et al., 2011b; Hanslmayr et al., 2014; Romei et al., 2015; see also Rosanova et al., 2009; Herring et al., 2015), analogous to tACS/otDCS. Yet, only one rTMS study on aftereffects (Weisz et al., 2014) optimized the design by tailoring stimulation frequency to underlying brain oscillations, which therefore may constitute another explanation of the discrepant patterns of rTMS vs. tACS/otDCS aftereffects. In addition, information about effects at stimulation frequency is often missing as several rTMS studies did not analyse aftereffects in this frequency band due to their focus on LTD and LTP-like effects. Finally, as most tACS/otDCS studies were designed to interact with oscillatory activity, experiments that failed to find frequency effects may be more likely to end up in the file drawer, i.e., might be more prone to publication bias. rTMS studies with different design rationales and hypotheses may suffer less from this particular problem.

Given that most of the reported rTMS aftereffects on brain oscillations are probably not driven by entrainment, could the classical dichotomy between excitatory and inhibitory rTMS [using high (>5 Hz) vs. low (< 1 Hz) rTMS frequencies, respectively] account for the variability in oscillatory aftereffects? This alternative explanation is also unlikely, given for instance that alpha-power enhancement after motor cortex stimulation has been observed across a wide variety of rTMS frequencies that cross the excitatory-inhibitory parameter divide (Veniero et al., 2011; see also Figure 1B). As recently highlighted by Huang et al. (2011), the classification of rTMS designs into high frequency/low frequency (or inhibitory/excitatory) protocols may also not be able to explain aftereffects on MEP amplitude. As for tACS/otDCS, it is therefore likely that the broadband aftereffects which seem to be independent from the stimulation frequency, might reflect LTP/LTP-like phenomena (see also Thickbroom, 2007), for which however a detailed mechanistic account is lacking. Huang et al. (2011) recently proposed a model that takes known cellular mechanisms of LTP/LTD into account to explain rTMS (specifically cTBS/iTBS) aftereffects on MEPs. This model shows that the pattern of stimulation (i.e., the interaction between number of pulses, intertrain interval, total stimulation time and the stimulation frequency) determines whether rTMS leads to a potentiation or depression of cortical excitability.

In light of the general lack of knowledge about effective rTMS targets (and by extension rTMS protocols) for controlled interventions, we believe it is worthwhile to design future rTMS studies to examine more explicitly the opportunity that direct interventions with brain oscillations may provide (see also Luber and Lisanby, 2014). This requires moving beyond the classical low vs. high frequency approach toward stricter frequency tuning of rTMS to underlying brain oscillations to allow comparisons between rTMS and oTDCS/tACS aftereffects.

### Gaps in existing evidence

Interest in brain oscillations as potential targets for NIBS has only recently gained momentum from studies demonstrating online entrainment (e.g., Fröhlich and McCormick, 2010; Reato et al., 2010; Thut et al., 2011b; Helfrich et al., 2014b). It is therefore unsurprising that our knowledge of possible oscillatory aftereffects, and optimal stimulation parameters for targeting specific oscillatory network activity, is still limited. In the following, we highlight what we believe are critical gaps in the existing evidence.

First, one largely unexplored aspect is the tailoring of the stimulation frequency to endogenous rhythms. The importance of a frequency match between external fields and underlying neural activity for maximum impact has been identified empirically in slice electrophysiology (Schmidt et al., 2014), and may be a crucial factor for guiding targeted interventions into intrinsic oscillatory activity. Yet, only six of the reviewed tACS/otDCS-studies tailored stimulation frequency to individual intrinsic frequencies (Zaehle et al., 2010; Neuling et al., 2013; Pahor and Jaušovec, 2014; Strüber et al., 2015; Vossen et al., 2015; Vosskuhl et al., 2015), vs. 16 tACS/otDCS-studies that did not take this factor into account. For rTMS, this ratio is even more unfavorable with one study (vs. 25) using frequency-tailored rTMS (Weisz et al., 2014). Note, however, that frequency matching alone does not guarantee aftereffects (Strüber et al., 2015), and that due to a tendency of intrinsic frequencies to fluctuate over time, deviations between stimulation frequency and intrinsic frequencies may affect the overall outcome (Vossen et al., 2015).

Second, there is often fragmentary coverage of frequency space, both in terms of applied stimulation and analyzed effect frequency. Many studies focus on one stimulation frequency (i.e., a control frequency is lacking) and limit their EEG/MEG analyses to one or few, broad frequency bands. This often effectively precludes the assessment of (sub-)harmonic changes that would be expected to be limited to narrow frequency bands.

Third, we note a substantial variability in stimulation montages/sites across studies, even for a given brain oscillation/network of interest. Especially for tACS/otDCS, little is known about optimal electrode sites and configurations, and how different montages might lead to different results with otherwise identical stimulation parameters. A systematic effort is required to compare different montages with respect to an intended manipulation, using guidance from modeling of effective field strength in the cortex (Dmochowski et al., 2011; Neuling et al., 2012b; Saturnino et al., 2015).

Fourth, besides the lack of information on associated behavioral changes discussed above, even with comparable external parameters the initial activation state of the cortex is likely to influence the system's response (state-dependency; e.g., Marshall et al., 2006; Kirov et al., 2009; Marshall et al., 2011; Neuling et al., 2013; Rizk et al., 2013) and will have to be taken into account. For example, aftereffects appear in different frequency bands when the same otDCS protocol is applied during NREM vs. REM sleep (Marshall et al., 2011), and alpha-tACS enhances alpha power only when participants have their eyes open but not closed (reflecting different baseline levels of alpha activity) (Neuling et al., 2013). Through mapping the state-dependency of stimulation outcomes, we might be able to improve NIBS efficacy, e.g., by capitalizing on closed loop approaches tracking fluctuations in specific ongoing spectral features to guide stimulation parameters (e.g., Brittain et al., 2013). For the latter, advances in algorithms for online tACS artifact correction are a prerequisite, as currently the analysis of EEG/MEG data obtained during stimulation can only be obtained offline (EEG: Helfrich et al., 2014b; for new developments with MEG see Garcia-Cossio et al., 2015; Neuling et al., 2015; Witkowski et al., 2015).

Fifth, inter- and intraindividual variability in the response to stimulation is a general and not trivial problem for NIBS studies and treatments (e.g., Ziemann and Siebner, 2015). Studies with large sample sizes and the assessment of multiple possible covariates are required. One aspect of intersubject variability is the different underlying anatomy. While TMS studies often benefit from neuronavigation using individual brain scans, this is not typically done in otDCS/tACS studies, but may lead to more reproducible outcomes. Intrasubject variability is even more difficult to tackle and would require multiple repeated sessions per participant using the same protocol (and simultaneous collection of state and trait variables) to get an idea of the extent (and causes) of such variability. Finally, more modeling efforts based on biologically plausible mechanisms of interventions are needed to better guide the exploration of the vast parameter space of tACS/otDCS and rTMS (Fröhlich, 2015).

Finally, we would like to point out that in order to map out the parameter space for protocols that produce desired changes in oscillatory activity it is important that investigators also publish their negative findings.

## Conclusion

While there is mounting evidence that oscillatory or rhythmic NIBS allows to control brain oscillations and associated functions during (online to) stimulation, we find mixed evidence for the controllability of NIBS-induced aftereffects on brain oscillations. We argue that while online mechanisms must be at the origin of offline effects, there is little evidence that the offline effects can be considered proxies of the online effects. Future studies will need to investigate the link between these online and offline effects to advance our understanding of rhythmic NIBS techniques and their potential for neuromodulation via controlled intervention into brain oscillations. Because a growing number of studies indicate that neurological and mental diseases are characterized by abnormal pattern of specific brain oscillations (the so called oscillopathies; Schnitzler and Gross, 2005; Light et al., 2006; Hammond et al., 2007; Edgar et al., 2015; Uhlhaas and Singer, 2015; for a review see Buzsáki and Watson, 2012), increasing our level of control over these aftereffect, and their direction and duration, will represent a decisive step to plan new strategies for neurorehabilitation.

## Funding

This work was supported by a Wellcome Trust Award to Gregor Thut and Joachim Gross [grant number 098434, 098433]. AV was supported by a PhD Studentship from the College of Science and Engineering, University of Glasgow.

### Conflict of interest statement

The authors declare that the research was conducted in the absence of any commercial or financial relationships that could be construed as a potential conflict of interest. The reviewer and handling Editor declared a current collaboration and the handling Editor states that the process nevertheless met the standards of a fair and objective review.
